# 3-(4-Hy­droxy-3-meth­oxy­phen­yl)acrylic acid–2,3,5,6-tetra­methyl­pyrazine (2/1)

**DOI:** 10.1107/S1600536811000961

**Published:** 2011-01-15

**Authors:** Zaiyou Tan, Erjia Zhu, Lin Luo, Zhuohui Lin, Ruisi Yan

**Affiliations:** a Department of Physical Chemistry, Guangdong Pharmaceutical University, Guangzhou, Guangdong 510006, People’s Republic of China

## Abstract

The asymmetric unit of the title compound, C_8_H_12_N_2_·2C_10_H_10_O_4_, contains a tetra­methyl­pyrazine mol­ecule, situated about an inversion center, and two substituted acrylic acid derivatives. The dihedral angle between the phenyl and pyrazine rings is 69.45 (9)°. In the crystal, inter­molecular O—H⋯O, O—H⋯N hydrogen bonds and weak C—H⋯O inter­actions lead to the formation of a supra­molecular network. The acrylic acid side chain is positionally disordered [occupancy ratio 0.852 (7):0.148 (7)].

## Related literature

For the synthesis of the title compound, see: Tan (2004 ▶). For the biological properties of the title compound, see: Tan *et al.* (2003 ▶).
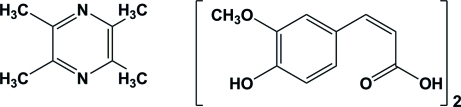


## Experimental

### 

#### Crystal data

0.5C_8_H_12_N_2_·C_10_H_10_O_4_
*M* *_r_* = 262.28
Monoclinic, 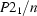

*a* = 9.4696 (7) Å
*b* = 5.7641 (4) Å
*c* = 24.3737 (15) Å
β = 93.654 (6)°
*V* = 1327.70 (16) Å^3^
*Z* = 4
Cu *K*α radiation
μ = 0.80 mm^−1^
*T* = 100 K
0.20 × 0.05 × 0.05 mm

#### Data collection

Oxford Diffraction Xcalibur Onyx Nova diffractometer
Absorption correction: multi-scan (*CrysAlis PRO*; Oxford Diffraction, 2009 ▶) *T*_min_ = 0.953, *T*_max_ = 0.961
4780 measured reflections
2398 independent reflections
2004 reflections with *I* > 2σi(*I*)
*R*_int_ = 0.030

#### Refinement

*R*[*F* ^2^ > 2σ(*F* ^2^)] = 0.054
*wR*(*F* ^2^) = 0.156
*S* = 1.09
2398 reflections
208 parameters
4 restraints
H-atom parameters constrained
Δρ_max_ = 0.63 e Å^−3^
Δρ_min_ = −0.40 e Å^−3^

### 

Data collection: *CrysAlis PRO* (Oxford Diffraction, 2009 ▶); cell refinement: *CrysAlis PRO*; data reduction: *CrysAlis PRO*; program(s) used to solve structure: *SHELXS97* (Sheldrick, 2008 ▶); program(s) used to refine structure: *SHELXL97* (Sheldrick, 2008 ▶); molecular graphics: *OLEX2* (Dolomanov *et al.*, 2009 ▶); software used to prepare material for publication: *OLEX2*.

## Supplementary Material

Crystal structure: contains datablocks I, global. DOI: 10.1107/S1600536811000961/su2237sup1.cif

Structure factors: contains datablocks I. DOI: 10.1107/S1600536811000961/su2237Isup2.hkl

Additional supplementary materials:  crystallographic information; 3D view; checkCIF report

## Figures and Tables

**Table 1:** Hydrogen-bond geometry (Å, °)

*D*—H⋯*A*	*D*—H	H⋯*A*	*D*⋯*A*	*D*—H⋯*A*
O2—H2⋯O1^i^	0.84	1.79	2.613 (5)	167
O4—H4⋯N1	0.82	1.97	2.749 (2)	158
C3—H3*B*⋯O4^ii^	0.98	2.58	3.553 (3)	174
C14—H14*B*⋯O4^ii^	0.98	2.59	3.465 (2)	148

Symmetry codes: (i) 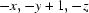; (ii) 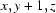.

**Fig. 1.**
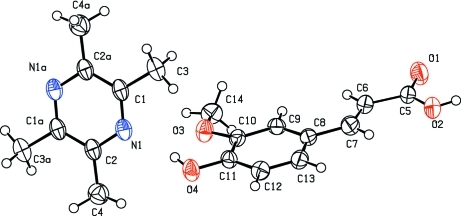
A view of the centrosymmmetric tetramethylpyrazine molecule and one of the ferulate molecules of the title compound. Displacement ellipsoids are drawn at the 50% probability level [Symmetry code: (a) = -x, -y, -z + 1; only the major component of the disordered acrylicacid side chain is shown].

## supplementary crystallographic information

### Comment

The title compound, tetramethylpyrazine ferulate is a pharmacologically significant compound which we found in some prescriptions of traditional Chinese medicine, and which are used to treat stroke patients. In both the invivo and invitro experiments, tetramethylpyrazine ferulate has a remarkable inhibitory effect on ADP induced platelet aggregation (Tan *et al.*, 2003). In order to study further its pharmacological effects the title compound was synthesized by the reaction of 3-(4-hydroxy-3-methoxyphenyl)-2-acrylicacid with tetrathylpyrazine, and its crystal structure is reported on herein.

X-ray crystallographic analysis confirmed the molecular structure and the atom connectivity for the title compound, as illustrated in Fig. 1. The dihedral angle between the mean planes of the pyrazine ring and phenyl ring (C8—C13) is 69.45 (9)°.

In the crystal intermolecular O—H···N hydrogen bonds and weak C—H···O interactions are observed, leading to the formation of a supra-molecular network (Table 1).

### Experimental

The title compound was synthesized according to the published procedure (Tan, 2004). Tetramethylpyrazine (2.8 g) was heated with 3-(4-hydroxy-3-methoxyphenyl)-2-acrylicacid (4.0 g) in acetone (45 ml). After refluxing at 333 K for 1 h, the reaction mixture was left to stand for several days, and yellow crystals were finally isolated.

### Refinement

The acid side chain is positionally disordered: occupancy of atoms C6/C6A, C/C5A, O1/O1A and O2/O2A were refined to be 0.852 (7)/0.148 (7). The following restraints were also applied: *DFIX* 1.32. 02 C7 C6 C7 C6A; *DFIX* 1.45. 02 C6 C5 C6A C5A; EADP C5 C5A. The OH and C-bound H-atoms were included in calculated positions and treated as riding atoms: O—H = 0.82 - 0.84 Å, C—H = 0.95 and 0.98Å for CH and CH_3_ H-atoms, respectively, with *U*_iso_(H) = k × *U*_eq_(C), where k = 1.5 for OH and CH_3_ H-atoms, and k = 1.2 for all other H-atoms.

### Crystal data

**Table:**

0.5C_8_H_12_N_2_·C_10_H_10_O_4_	*F*(000) = 556
*M**_r_* = 262.28	*D*_x_ = 1.312 Mg m^−^^3^
Monoclinic, *P*2_1_/*n*	Cu *K*α radiation, λ = 1.54180 Å
Hall symbol: -P 2yn	Cell parameters from 2625 reflections
*a* = 9.4696 (7) Å	θ = 3.6–71.3°
*b* = 5.7641 (4) Å	µ = 0.80 mm^−^^1^
*c* = 24.3737 (15) Å	*T* = 100 K
β = 93.654 (6)°	Plate, yellow
*V* = 1327.70 (16) Å^3^	0.20 × 0.05 × 0.05 mm
*Z* = 4	

### Data collection

**Table:**

Oxford Diffraction Xcalibur Onyx Nova diffractometer	2398 independent reflections
Radiation source: fine-focus sealed tube	2004 reflections with *I* > 2σi(*I*)
graphite	*R*_int_ = 0.030
Detector resolution: 8.2417 pixels mm^-1^	θ_max_ = 68.2°, θ_min_ = 3.6°
ω scans	*h* = −6→11
Absorption correction: multi-scan (*CrysAlis PRO*; Oxford Diffraction, 2009)	*k* = −6→6
*T*_min_ = 0.953, *T*_max_ = 0.961	*l* = −29→29
4780 measured reflections	

### Refinement

**Table:**

Refinement on *F*^2^	Primary atom site location: structure-invariant direct methods
Least-squares matrix: full	Secondary atom site location: difference Fourier map
*R*[*F*^2^ > 2σ(*F*^2^)] = 0.054	Hydrogen site location: inferred from neighbouring sites
*wR*(*F*^2^) = 0.156	H-atom parameters constrained
*S* = 1.09	*w* = 1/[σ^2^(*F*_o_^2^) + (0.0848*P*)^2^ + 0.4876*P*] where *P* = (*F*_o_^2^ + 2*F*_c_^2^)/3
2398 reflections	(Δ/σ)_max_ = 0.003
208 parameters	Δρ_max_ = 0.63 e Å^−^^3^
4 restraints	Δρ_min_ = −0.40 e Å^−^^3^

### Special details

**Table:**

Experimental. CrysAlisPro (Oxford Diffraction, 2009) Empirical absorption correction using spherical harmonics, implemented in SCALE3 ABSPACK scaling algorithm.
Geometry. Bond distances, angles *etc*. have been calculated using the rounded fractional coordinates. All su's are estimated from the variances of the (full) variance-covariance matrix. The cell e.s.d.'s are taken into account in the estimation of distances, angles and torsion angles
Refinement. Refinement of *F*^2^ against ALL reflections. The weighted *R*-factor *wR* and goodness of fit *S* are based on *F*^2^, conventional *R*-factors *R* are based on *F*, with *F* set to zero for negative *F*^2^. The threshold expression of *F*^2^ > σ(*F*^2^) is used only for calculating *R*-factors(gt) *etc*. and is not relevant to the choice of reflections for refinement. *R*-factors based on *F*^2^ are statistically about twice as large as those based on *F*, and *R*- factors based on ALL data will be even larger.

### Fractional atomic coordinates and isotropic or equivalent isotropic displacement parameters (Å^2^)

**Table:**

	*x*	*y*	*z*	*U*_iso_*/*U*_eq_	Occ. (<1)
O1	−0.0744 (5)	0.5740 (6)	0.06036 (17)	0.0523 (10)	0.852 (7)
O2	0.0563 (4)	0.2666 (5)	0.03930 (15)	0.0489 (9)	0.852 (7)
O3	−0.11323 (15)	0.0544 (3)	0.33773 (5)	0.0432 (4)	
O4	0.04554 (15)	−0.3292 (2)	0.35223 (6)	0.0444 (5)	
C5	−0.0143 (5)	0.3836 (9)	0.07299 (14)	0.0391 (11)	0.852 (7)
C6	−0.0257 (2)	0.2939 (4)	0.12865 (9)	0.0392 (7)	0.852 (7)
C7	0.0333 (2)	0.1014 (4)	0.14611 (9)	0.0506 (7)	
C8	0.0286 (2)	−0.0041 (4)	0.20015 (8)	0.0404 (6)	
C9	−0.0448 (2)	0.0919 (3)	0.24314 (8)	0.0375 (6)	
C10	−0.04259 (19)	−0.0175 (3)	0.29389 (7)	0.0334 (5)	
C11	0.03695 (19)	−0.2208 (3)	0.30294 (7)	0.0347 (5)	
C12	0.1057 (2)	−0.3180 (4)	0.26015 (8)	0.0395 (6)	
C13	0.1010 (2)	−0.2104 (4)	0.20929 (8)	0.0416 (6)	
C14	−0.2088 (2)	0.2438 (4)	0.32912 (9)	0.0462 (7)	
O1A	−0.051 (2)	0.484 (4)	0.0621 (8)	0.045 (6)	0.148 (7)
O2A	0.1074 (19)	0.249 (3)	0.0201 (7)	0.054 (5)	0.148 (7)
C5A	0.028 (3)	0.322 (5)	0.0629 (11)	0.0391 (11)	0.148 (7)
C6A	0.0587 (13)	0.162 (2)	0.1065 (5)	0.038 (4)	0.148 (7)
N1	0.00905 (17)	−0.1036 (3)	0.44970 (6)	0.0393 (5)	
C1	0.0891 (2)	0.0814 (4)	0.46363 (8)	0.0383 (6)	
C2	−0.0802 (2)	−0.1871 (3)	0.48531 (8)	0.0378 (6)	
C3	0.1847 (2)	0.1711 (4)	0.42178 (9)	0.0479 (7)	
C4	−0.1659 (2)	−0.3952 (4)	0.46761 (10)	0.0503 (7)	
H4	0.01920	−0.24080	0.37590	0.0670*	
H6	−0.07890	0.38050	0.15330	0.0470*	0.852 (7)
H2	0.05430	0.33540	0.00890	0.0730*	0.852 (7)
H14A	−0.27540	0.20980	0.29770	0.0690*	
H14B	−0.15560	0.38480	0.32160	0.0690*	
H14C	−0.26120	0.26690	0.36210	0.0690*	
H7	0.08590	0.01970	0.12040	0.0610*	0.852 (7)
H9	−0.09610	0.23230	0.23740	0.0450*	
H12	0.15630	−0.45930	0.26570	0.0470*	
H13	0.14830	−0.27930	0.18020	0.0500*	
H6AA	0.13470	0.06810	0.09570	0.0450*	0.148 (7)
H6AB	−0.05690	0.17360	0.14590	0.0610*	0.148 (7)
H2A	0.09850	0.34570	−0.00580	0.0810*	0.148 (7)
H3A	0.20300	0.04760	0.39550	0.0720*	
H3B	0.13940	0.30260	0.40220	0.0720*	
H3C	0.27440	0.22170	0.44030	0.0720*	
H4A	−0.12420	−0.46860	0.43620	0.0750*	
H4B	−0.26310	−0.34730	0.45700	0.0750*	
H4C	−0.16650	−0.50610	0.49810	0.0750*	

### Atomic displacement parameters (Å^2^)

**Table:**

	*U*^11^	*U*^22^	*U*^33^	*U*^12^	*U*^13^	*U*^23^
O1	0.0654 (19)	0.053 (2)	0.0387 (12)	0.0041 (16)	0.0046 (11)	0.0104 (15)
O2	0.0630 (18)	0.0519 (12)	0.0320 (15)	−0.0025 (11)	0.0044 (11)	0.0042 (11)
O3	0.0487 (8)	0.0467 (8)	0.0339 (7)	0.0155 (6)	0.0009 (6)	0.0038 (6)
O4	0.0586 (9)	0.0409 (8)	0.0337 (7)	0.0124 (6)	0.0032 (6)	0.0103 (6)
C5	0.046 (2)	0.045 (2)	0.0266 (16)	−0.0126 (18)	0.0039 (12)	−0.0014 (14)
C6	0.0447 (13)	0.0429 (13)	0.0302 (12)	−0.0046 (10)	0.0036 (9)	−0.0014 (9)
C7	0.0541 (12)	0.0586 (14)	0.0381 (12)	−0.0193 (11)	−0.0043 (9)	0.0034 (10)
C8	0.0428 (10)	0.0473 (11)	0.0304 (9)	−0.0178 (9)	−0.0024 (8)	0.0015 (8)
C9	0.0425 (10)	0.0327 (9)	0.0357 (10)	−0.0048 (8)	−0.0090 (8)	0.0055 (7)
C10	0.0367 (9)	0.0343 (9)	0.0286 (9)	0.0002 (7)	−0.0027 (7)	0.0003 (7)
C11	0.0366 (9)	0.0357 (10)	0.0312 (9)	−0.0006 (7)	−0.0018 (7)	0.0046 (7)
C12	0.0372 (10)	0.0422 (11)	0.0389 (10)	0.0010 (8)	0.0017 (8)	−0.0008 (8)
C13	0.0409 (10)	0.0502 (12)	0.0339 (10)	−0.0076 (9)	0.0043 (8)	−0.0041 (8)
C14	0.0463 (11)	0.0405 (11)	0.0516 (12)	0.0110 (9)	0.0011 (9)	−0.0011 (9)
O1A	0.054 (10)	0.050 (15)	0.034 (7)	0.014 (10)	0.025 (7)	0.024 (10)
O2A	0.071 (10)	0.057 (7)	0.035 (7)	0.008 (7)	0.011 (6)	0.015 (6)
C5A	0.046 (2)	0.045 (2)	0.0266 (16)	−0.0126 (18)	0.0039 (12)	−0.0014 (14)
C6A	0.037 (7)	0.042 (8)	0.034 (8)	0.011 (5)	0.003 (5)	0.012 (6)
N1	0.0404 (8)	0.0450 (9)	0.0322 (8)	0.0081 (7)	−0.0007 (7)	0.0108 (7)
C1	0.0365 (9)	0.0450 (11)	0.0331 (9)	0.0087 (8)	0.0010 (7)	0.0140 (8)
C2	0.0356 (9)	0.0446 (11)	0.0327 (9)	0.0072 (8)	−0.0007 (7)	0.0122 (8)
C3	0.0478 (11)	0.0549 (13)	0.0420 (11)	0.0058 (9)	0.0107 (9)	0.0151 (9)
C4	0.0486 (12)	0.0520 (13)	0.0498 (12)	−0.0011 (10)	0.0002 (10)	0.0051 (10)

### Geometric parameters (Å, °)

**Table:**

O1—C5	1.265 (6)	C11—C12	1.383 (3)
O1A—C5A	1.20 (4)	C12—C13	1.384 (3)
O2—C5	1.283 (6)	C6—H6	0.9500
O2A—C5A	1.39 (3)	C6A—H6AA	0.9500
O3—C14	1.425 (3)	C7—H7	0.9500
O3—C10	1.361 (2)	C7—H6AB	0.9500
O4—C11	1.352 (2)	C9—H9	0.9500
O2—H2	0.8400	C12—H12	0.9500
O2A—H2A	0.8400	C13—H13	0.9500
O4—H4	0.8200	C14—H14B	0.9800
N1—C1	1.340 (3)	C14—H14C	0.9800
N1—C2	1.339 (2)	C14—H14A	0.9800
C5—C6	1.462 (4)	C1—C3	1.498 (3)
C5A—C6A	1.42 (3)	C1—C2^i^	1.393 (3)
C6—C7	1.302 (3)	C2—C4	1.496 (3)
C6A—C7	1.068 (12)	C3—H3A	0.9800
C7—C8	1.454 (3)	C3—H3B	0.9800
C8—C13	1.384 (3)	C3—H3C	0.9800
C8—C9	1.407 (3)	C4—H4A	0.9800
C9—C10	1.387 (3)	C4—H4B	0.9800
C10—C11	1.403 (2)	C4—H4C	0.9800
			
C10—O3—C14	117.19 (15)	C8—C7—H7	116.00
C5—O2—H2	109.00	C8—C7—H6AB	96.00
C5A—O2A—H2A	109.00	C10—C9—H9	120.00
C11—O4—H4	110.00	C8—C9—H9	120.00
C1—N1—C2	119.49 (16)	C13—C12—H12	120.00
O2—C5—C6	118.7 (4)	C11—C12—H12	120.00
O1—C5—C6	118.3 (4)	C8—C13—H13	119.00
O1—C5—O2	123.0 (4)	C12—C13—H13	119.00
O1A—C5A—O2A	126 (2)	H14A—C14—H14C	109.00
O2A—C5A—C6A	106 (2)	H14B—C14—H14C	110.00
O1A—C5A—C6A	128 (2)	H14A—C14—H14B	109.00
C5—C6—C7	123.3 (3)	O3—C14—H14A	109.00
C5A—C6A—C7	147.1 (16)	O3—C14—H14B	109.00
C6A—C7—C8	167.7 (7)	O3—C14—H14C	109.00
C6—C7—C8	128.0 (2)	N1—C1—C3	117.31 (18)
C9—C8—C13	118.77 (18)	N1—C1—C2^i^	120.61 (17)
C7—C8—C13	117.51 (18)	C2^i^—C1—C3	122.06 (19)
C7—C8—C9	123.72 (19)	N1—C2—C4	117.03 (17)
C8—C9—C10	120.34 (17)	N1—C2—C1^i^	119.90 (17)
O3—C10—C9	125.55 (16)	C1^i^—C2—C4	123.07 (18)
O3—C10—C11	114.63 (15)	C1—C3—H3A	110.00
C9—C10—C11	119.82 (16)	C1—C3—H3B	109.00
O4—C11—C12	118.58 (16)	C1—C3—H3C	109.00
C10—C11—C12	119.59 (17)	H3A—C3—H3B	109.00
O4—C11—C10	121.81 (15)	H3A—C3—H3C	109.00
C11—C12—C13	120.3 (2)	H3B—C3—H3C	109.00
C8—C13—C12	121.10 (19)	C2—C4—H4A	109.00
C7—C6—H6	118.00	C2—C4—H4B	109.00
C5—C6—H6	118.00	C2—C4—H4C	109.00
C5A—C6A—H6AA	107.00	H4A—C4—H4B	110.00
C7—C6A—H6AA	106.00	H4A—C4—H4C	109.00
C6—C7—H7	116.00	H4B—C4—H4C	109.00
C6A—C7—H6AB	96.00		
			
C14—O3—C10—C9	6.9 (3)	C7—C8—C13—C12	177.84 (19)
C14—O3—C10—C11	−172.83 (16)	C8—C9—C10—C11	2.1 (3)
C1—N1—C2—C4	−179.50 (18)	C8—C9—C10—O3	−177.59 (18)
C2—N1—C1—C2^i^	0.0 (3)	O3—C10—C11—O4	−2.3 (3)
C1—N1—C2—C1^i^	0.0 (3)	O3—C10—C11—C12	175.86 (17)
C2—N1—C1—C3	−178.63 (17)	C9—C10—C11—O4	177.98 (17)
O2—C5—C6—C7	0.6 (6)	C9—C10—C11—C12	−3.8 (3)
O1—C5—C6—C7	−179.2 (4)	C10—C11—C12—C13	2.7 (3)
C5—C6—C7—C8	−179.8 (3)	O4—C11—C12—C13	−179.10 (17)
C6—C7—C8—C9	0.3 (3)	C11—C12—C13—C8	0.3 (3)
C6—C7—C8—C13	−179.6 (2)	N1—C1—C2^i^—N1^i^	0.0 (3)
C7—C8—C9—C10	−179.05 (18)	N1—C1—C2^i^—C4^i^	−179.47 (18)
C9—C8—C13—C12	−2.1 (3)	C3—C1—C2^i^—N1^i^	178.56 (18)
C13—C8—C9—C10	0.9 (3)	C3—C1—C2^i^—C4^i^	−0.9 (3)

Symmetry codes: (i) −*x*, −*y*, −*z*+1.

### Hydrogen-bond geometry (Å, °)

**Table:**

*D*—H···*A*	*D*—H	H···*A*	*D*···*A*	*D*—H···*A*
O2—H2···O1^ii^	0.84	1.79	2.613 (5)	167
O4—H4···O3	0.82	2.28	2.685 (2)	111
O4—H4···N1	0.82	1.97	2.749 (2)	158
C3—H3B···O4^iii^	0.98	2.58	3.553 (3)	174
C14—H14B···O4^iii^	0.98	2.59	3.465 (2)	148

Symmetry codes: (ii) −*x*, −*y*+1, −*z*; (iii) *x*, *y*+1, *z*.

## References

[bb1] Dolomanov, O. V., Bourhis, L. J., Gildea, R. J., Howard, J. A. K. & Puschmann, H. (2009). *J. Appl. Cryst.* **42**, 339–341.

[bb2] Oxford Diffraction (2009). *CrysAlis PRO* Oxford Diffraction Ltd, Yarnton, England.

[bb3] Sheldrick, G. M. (2008). *Acta Cryst.* A**64**, 112–122. 10.1107/S0108767307043930 18156677

[bb4] Tan, Z. (2004). China Patent No. ZL00114239.9.

[bb5] Tan, Z., Jiang, T., Tang, C., Luo, J., Tan, H. & Chen, R. (2003). *J. Chin. New Drugs*, **12**, 529-531.

